# Biohydrogen production from enzymatic hydrolysis of food waste in batch and continuous systems

**DOI:** 10.1038/srep38395

**Published:** 2016-12-02

**Authors:** Wei Han, Yingting Yan, Yiwen Shi, Jingjing Gu, Junhong Tang, Hongting Zhao

**Affiliations:** 1 College of Materials and Environmental Engineering, Hangzhou Dianzi University, Hangzhou 310018, China

## Abstract

In this study, the feasibility of biohydrogen production from enzymatic hydrolysis of food waste was investigated. Food waste (solid-to-liquid ratio of 10%, w/v) was first hydrolyzed by commercial glucoamylase to release glucose (24.35 g/L) in the food waste hydrolysate. Then, the obtained food waste hydrolysate was used as substrate for biohydrogen production in the batch and continuous (continuous stirred tank reactor, CSTR) systems. It was observed that the maximum cumulative hydrogen production of 5850 mL was achieved with a yield of 245.7 mL hydrogen/g glucose (1.97 mol hydrogen/mol glucose) in the batch system. In the continuous system, the effect of hydraulic retention time (HRT) on biohydrogen production from food waste hydrolysate was investigated. The optimal HRT obtained from this study was 6 h with the highest hydrogen production rate of 8.02 mmol/(h·L). Ethanol and acetate were the major soluble microbial products with low propionate production at all HRTs. Enzymatic hydrolysis of food waste could effectively accelerate hydrolysis speed, improve substrate utilization rate and increase hydrogen yield.

Due to environmental pollution and gradual deletion of fossil fuels, the development of clean and sustainable energy has attracted great attentions in the last decades^1^. Hydrogen is considered to be one of the most promising future energy carriers because it is renewable and produces only water when combusted^2^. Furthermore, the energy yield of hydrogen is 122 kJ/g which is 2.75 times higher that of fossil fuel^3^. Generally, hydrogen production could be achieved in physicochemical and biological processes^4^. Conventional physicochemical processes (such as steam reforming of hydrocarbons and coal gasification) are neither sustainable nor environmental friendly because fossil fuels are used as substrate. In contrast, biological processes seem to be more attractive because a wide variety of organic waste materials could be used as substrate and the processes could be operated under room temperature and pressure conditions^5^,^6^. In particular, dark fermentative hydrogen production is regarded as a more feasible commercial process since it could achieve high hydrogen production rate without the limitation of light^7^.

Dark fermentation could utilize a wide range of organic waste or wastewater as carbon source for hydrogen production. Ren *et al*.^8^, investigated the feasibility of biohydrogen production from molasses and found the optimal hydrogen yield of 98.7 mmol/L in the up-flow anaerobic sludge blanket. Han *et al*.^9^, used waste wheat as substrate and achieved the maximum hydrogen yield of 106.23 mmol/L in the batch system. Food waste could be a promising carbon source for dark fermentative hydrogen production since it could reduce hydrogen production cost and recycle the organic municipal solid waste^10^. However, it is difficult to directly use food waste as feedstock for biohydrogen production because the nutrients stored in the food waste are in the form of starch which has to be converted into glucose before used by hydrogen-producing microorganisms^11^. Meanwhile, the hydrolysis is considered to be the limiting step for biohydrogen production from food waste^12^. It has been reported that chemical pretreatment could hydrolyze the macromolecule into micromolecule, but the inhibitors (such as furfural) for further biohydrogen production could be also produced^13^. Enzymatic hydrolysis, which is able to degrade starch contained in the food waste into glucose with advantage of common conditions and no inhibitors production, could be a promising way^14^. And, the glucose from food waste has been used as substrate for a variety of fermentative productions. such as lactic acid^15^ and succinic acid^16^. However, information about biohydrogen production from enzymatic hydrolysis of food waste is limited.

Therefore, a two-stage bioprocess for hydrogen production from food waste was developed in this study. Food waste was first hydrolyzed by glucoamylse to produce food waste hydrolysate. Then, the food waste hydrolysate was used as substrate for biohydrogen production in the batch (fermenter) and continuous (continuous stirred tank reactor, CSTR) systems. The feasibility of biohydrogen production from food waste hydrolysate in the fermentor and the effect of hydraulic retention time (HRT) on biohydrogen production in the CSTR were also investigated, respectively.

## Results and Discussion

### Enzymatic hydrolysis of food waste by the commercial glucoamylase

**Figure 1:**
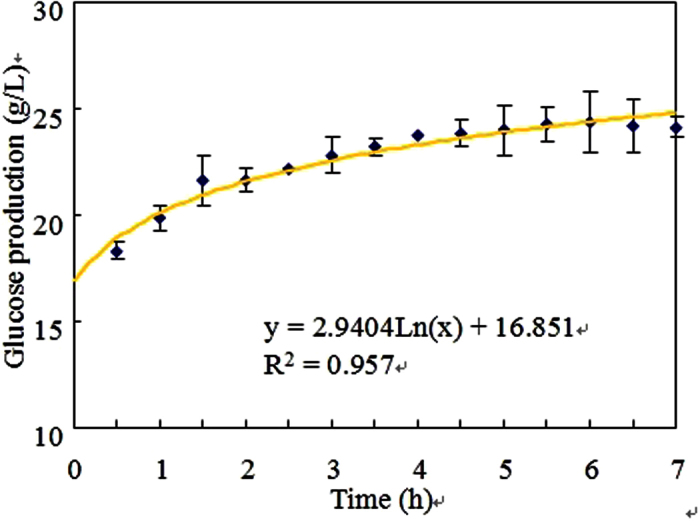
Glucose production in the enzymatic hydrolysis of food waste.

The pretreated food waste was hydrolyzed by the commercial glucoamylase and the glucose production was shown in Fig. 1. It was observed that glucose could be released from food waste via enzymatic hydrolysis and increased with time. Linear regression result showed that the correlation between glucose production (y) and time (x) could be expressed as y = 2.9404Ln(x) + 16.851 (R^2^ = 0.957). The maximum glucose production of 24.35 g/L was achieved in the food waste hydrolysate. Hydrolysis and liquification are considered to be the limiting step for biohydrogen production from food waste. In this study, food waste with solid-to-liquid ratio of 10% (w/v) could release 24.35 g/L glucose via enzymatic hydrolysis within 7 h.

The food waste used in this study consisted of around 406 mg starch/g food waste. According to the molar basis of starch hydrolysis, the theoretical glucose production of 451.1 mg glucose/g food waste could be calculated. In this study, around 304.4 mg glucose/g food waste could be produced via enzymatic hydrolysis. The starch conversion efficiency of food waste could reach 67.5%. So, it was concluded that enzymatic hydrolysis of food waste could effectively accelerate the hydrolysis speed and liquefy solid food waste into liquid food waste hydrolysate.

As shown in Table 1, the glucose production and yield in the food waste hydrolysate by commercial glucoamylase were lower than using combined enzymes produced from solid state fermentation by fungi (*Aspergillus awamori* and *Aspergillus oryzae*)^17^. This is probably because the combined enzymes contained other glucose-producing enzymes (such as α-amylase and lactase) which could digest specific components of food waste into glucose. For example, long-chain carbohydrates could be broken into glucose or maltose by α-amylase. However, the hydrolysis time by commercial enzyme was only 7 h which was much shorter than using combined enzymes produced from solid state fermentation by fungi. It was important for industrial biohydrogen production from food waste because the shorter hydrolysis time could effectively reduce the hydrogen production cost.

### Biohydrogen production from food waste hydrolysate in the batch system

#### Cumulative hydrogen production and glucose utilization

**Figure 2:**
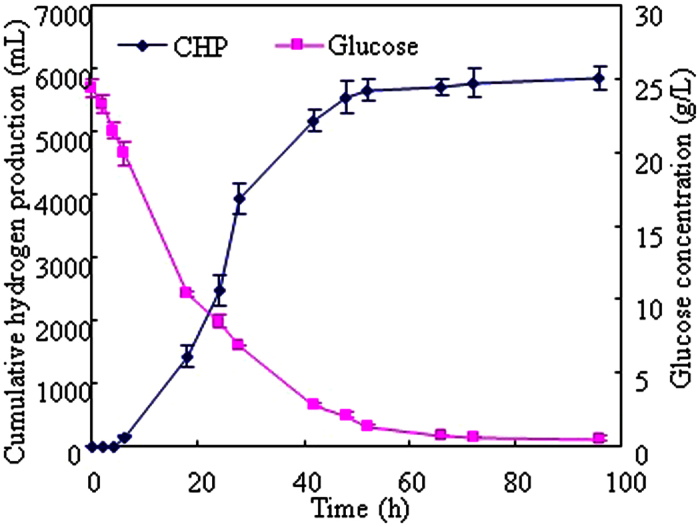
Cumulative hydrogen production from food waste hydrolysate in the batch system.

Biohydrogen production from food waste hydrolysate in the batch system was investigated in this section. Figure 2 showed the fermentation profiles for cumulative hydrogen production (CHP) and glucose utilization. A significant increasing of CHP from 0 mL to 5550 mL and decreasing of glucose concentration from 24.35 g/L to 2.12 g/L was observed within 48 h which indicated that the food waste hydrolysate contained sufficient nutrients for biohydrogen production. At last, around 5850 mL hydrogen was produced and 23.81 g glucose was consumed after 96 h which corresponded to a yield of 245.7 mL hydrogen/g glucose (1.97 mol hydrogen/mol glucose).

A modified Gompertz equation (1) was used to simulate biohydrogen production from food waste hydrolysate and the constant was determined by regression analysis using the Matlab 8.0 program.


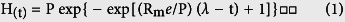


where, H(t) is the CHP (mL), P is the maximum hydrogen production potential (mL), R_m_ is the maximum hydrogen production rate (mL/h), λ is duration of the lag phase, *e* is 2.718 and *t* is the cultivation time (h). According to Gompertz equation, the maximum hydrogen production rate of 277.8 mL/(h), the maximum hydrogen production potential (P) of 6437 mL and the lag phase of 11.2 h could be calculated.

#### Soluble microbial products and carbon recovery

Table 2 showed the profile of soluble microbial products and carbon recovery from food waste hydrolysate. The main soluble microbial products were ethanol and acetate with yields of 108.93 mmol and 79.4 mmol, respectively. It indicated that the bacterial metabolism was following ethanol type fermentation. This was beneficial for biohydrogen production because the ethanol type fermentation was considered to be the best type for biohydrogen production^18^.

According to the carbon produced in the ethanol, acetate, butyrate and carbon dioxide, the recovered carbon in the soluble and gaseous microbial products of 665.45 mmol could be calculated. The consumed glucose was 132.27 mmol which was equal to consumed carbon of 793.62 mmol. Therefore, the carbon recovery of 83.8% was calculated in the batch system with the balance assumed to be converted to biomass^19^.

### Biohydrogen production from food waste hydrolysate with various HRT in the continuous system

#### Biohydrogen production rate and biomass

**Figure 3:**
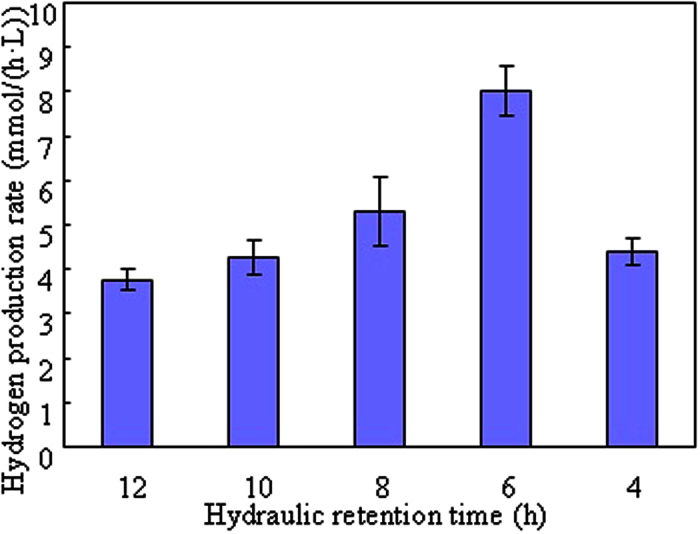
Hydrogen production rate in the CSTR from food waste hydrolysate.

**Figure 4:**
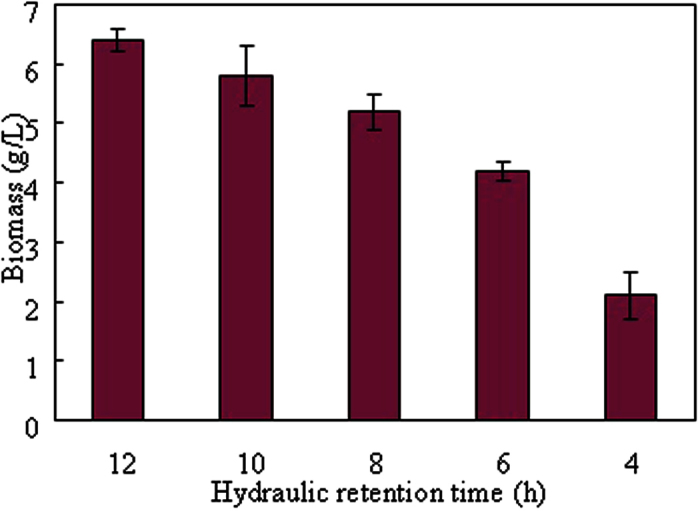
Biomass concentrations with different HRTs from food waste hydrolysate in the CSTR.

Hydrogen production rate (HPR) is a key criterion to evaluate the performance of biohydrogen-producing system. The HPRs at different HRT conditions (4–12 h) from food waste hydrolysate in the CSTR were shown in Fig. 3. For HRTs between 12 and 6 h, the HPR increased from 3.76 mmol/(h·L) to 8.02 mmol/(h·L) with decreasing of HRT since much more organic substrate was supplied into the CSTR for biohydrogen production^20^. However, the HPR decreased to 4.4 mmol/(h·L) when the HRT further decreased to 4 h. This is probably because the low HRT (4 h) had a direct negative effect on the hydrogen-producing microorganisms which resulted in the decrease of HPR. Moreover, it was observed in Fig. 4 that a severe loss of biomass was happened at HRT of 4 h because the shorter HRT (4 h) could not provide a favorable condition for remaining hydrogen-producing microorganisms^21^. Therefore, it was concluded that the optimal HRT for HPR in this study was 6 h with the highest HPR of 8.02 mmol/(h·L). The generated biogas was composed of hydrogen and carbon dioxide. Methane was not detected throughout the whole study which supported the effectiveness of heat pretreatment on seed sludge.

The comparison of HPR obtained from this study with other reported studies was shown in Table 3. Using glucose as substrate in a fix-bed reactor, Wu *et al*.^22^ got the optimal HPR of 16.1 mmol/(h·L) with HRT of 4 h. Zhao *et al*.^23^ investigated the effect of HRT on HPR from glucose and xylose in the UASB and found that the maximum HPR of 5.4 mmol/(h·L) was achieved with HRT of 12 h. It was observed that the HPR obtained from this study was comparable or higher than the reported studies. However, the results obtained from this study seemed to be more attractive for industrial application because the substrate used in this study was food waste hydrolysate rather than glucose.

#### Soluble microbial product, glucose utilization and carbon recovery

The soluble microbial products (SMPs) produced in the CSTR with various HRTs were shown in Table 4. Similar to the HPR, the SMP increased from 25.43 mmol/L to 36.87 mmol/L when the HRT decreased from 12 h to 6 h and decreased to 20.82 mmol/L with HRT further decreased to 4 h. It was observed that ethanol and acetate were the major SMPs with low propionate production at all HRTs in the CSTR. This was beneficial for biohydrogen production since ethanol and acetate productions were in general positively correlated to biohydrogen production, Eqs (2) and (3) 24, whereas propionate production consumed free electron derived from NADH, thereby being unfavorable to biohydrogen production, Eq. (4) 25.


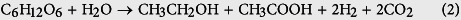



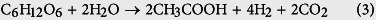



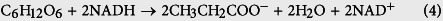


Table 5 showed the glucose utilizations ad carbon recoveries with various HRTs in the CSTR. It was found that the glucose utilization was around 92.5–98.2% in the CSTR at HRT = 12–6 h, indicating an efficient substrate utilization under those conditions. Reduction of HRT to 4 h resulted in a drastic decrease of glucose utilization (60.7%) which could be attributed to the washout of hydrogen-producing sludge (Fig. 4). The carbon recovery ranged from 67.3% to 86.9% at various HRT with the balance assumed to be the growth of biomass.

### Carbon and material balances of biohydrogen production from food waste in the batch and continuous systems

**Figure 5:**
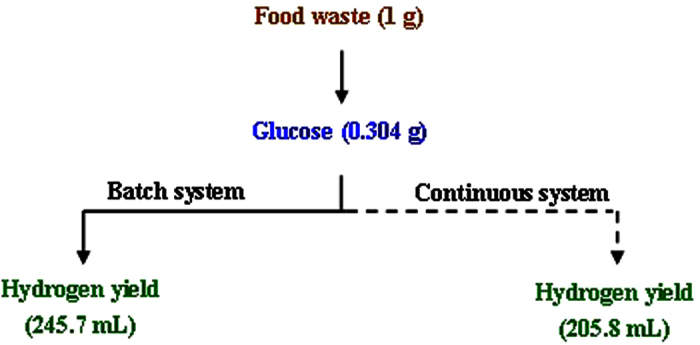
Material balance of biohydrogen production from enzymatic hydrolysis of food waste in the batch and continuous systems.

The carbon balance of biohydrogen production from food waste hydrolysate in the CSTR with the optimal HRT (6 h) was shown Table 6. It was observed that the carbon content of the food waste included 32.5% for undigested food waste and 67.5% for carbon dioxide and SMP productions. The ethanol (15.7–18.52%) and carbon dioxide (13.74–27.88%) accounted for the largest parts of consumed carbon since they were the main soluble and gaseous products. According to the enzymatic hydrolysis of food waste (Fig. 1) and biohydrogen production from food waste hydrolysate in the batch and continuous systems (Figs 2 and 3), it could be calculated that 1 g food waste could produce 0.304 g glucose in the food waste hydrolysate. Then, it could be further converted to 245.7 mL (1380 mL/g VSS_added_) in the batch system or 205.8 mL (1156 mL/g VSS_added_) hydrogen in the continuous system (Fig. 5). It was found that the hydrogen yield obtained in the batch system was higher than in the continuous system probably the glucose could also be washed out with effluent and have a negative influence on hydrogen yield in the continuous mode^26^.

As shown in Table 7, the hydrogen yields obtained from the batch and continuous systems were 967.2 mL/g VSS_added_ and 810.2 mL/g VSS_added_, respectively, which were obviously higher than other reported studies (Table 7). This was because the substrate used in this study was food waste hydrolysate rather than solid food waste and no inhibitive by-products for further biohydrogen production were produced in enzymatic hydrolysis. And, the oil in the food waste, which had a big negative influence on hydrogen-producing sludge, had been removed by the proposed process. Therefore, it was concluded that enzymatic hydrolysis of food waste could effectively accelerate hydrolysis speed, improve substrate utilization rate and increase hydrogen yield.

## Conclusions

In this study, enzymatic hydrolysis of food waste was used as substrate for biohydrogen production in the batch and continuous systems.

1. Food waste could release 24.35 g/L glucose via enzymatic hydrolysis within 7 h. The starch conversion efficiency of food waste could reach 67.5%. Enzymatic hydrolysis of food waste could effectively accelerate the hydrolysis speed and liquefy solid food waste into liquid food waste hydrolysate.
2. In the batch system, around 58550 mL hydrogen was produced and 23.81 g glucose was consumed after 96 h which corresponded to a yield of 245.7 mL hydrogen/g glucose (1.97 mol hydrogen/mol glucose). While, in the continuous system, the optimal HRT for HPR in this study was 6 h with the highest HPR of 8.02 mmol/(h·L). The ethanol (15.7–18.52%) and carbon dioxide (13.74–27.88%) accounted for the largest parts of consumed carbon since they were the main soluble and gaseous products. The hydrogen yield obtained in the continuous system (245.7 mL) was higher than in the batch system (205.8 mL) because the wash-out of glucose with effluent in the continuous mode.

## Material and Methods

### Feedstock, commercial enzyme and seed sludge

The food waste used in this study was collected from a university canteen (Hangzhou Dianzi University, China). Prior to enzymatic hydrolysis, the collected food waste was cut into smaller physical size by the kitchen blender to improve the hydrolysis efficiency. The composition of food waste was measured according to Standard Method^27^ and listed in Table 8.

The commercial glucoamylase, which was purchased from Shanghai Beinuo Biotechnology Co., Ltd., was utilized in enzymatic hydrolysis of food waste. The activity of glucoamylase was specified to be 2000 U/g by the supplier, in which 1 U was defined as the amount of enzyme hydrolyzing 1 g of starch/h at 40 °C and pH 6.

The seed sludge was provided by a local municipal wastewater treatment plant (Zhi Jiang Wastewater Treatment Plant, China). It was pretreated by heating in a water bath at 100 °C for 6 h to remove methanogenic bacteria and then used as inoculum for biohydrogen production.

### Enzymatic hydrolysis of food waste

Enzymatic hydrolysis of food waste was carried out in a bioreactor with working volume of 1 L. The ground food waste was added into the bioreactor and diluted with tap water to a solid-to-liquid ratio of 10% (w/v). The commercial glucoamylase was then inoculated in the bioreactor when the temperature and agitation speed reached 55 °C and 500 rpm, respectively. Samples were withdrawn in half an hour to measure the production of glucose. When the glucose concentration stopped increasing, the enzymatic hydrolysis was done and the mixture was centrifuged at 10,000 rpm for 30 min and filtered by Whatman No. 1 filter paper to achieve the liquid food waste hydrolysate which was used as substrate for further biohydrogen production. It was important to note that the oil could be also removed by this step.

### Biohydrogen production from food waste hydrolysate in the batch and continuous systems

In this study, the food waste hydrolysate was used as substrate for biohydrogen production in the batch and continuous systems. For the batch system, biohydrogen production was carried out in a fermentor with working volume of 1 L. External nitrogen gas was sparged into the fermentor at a rate of 0.5 vvm for 10 min to provide the anaerobic condition for biohydrogen production. The agitation speed and temperature of the fermentor was set to be 200 rpm and 37 °C, respectively. The continuous experiments were performed in a continuous stirred tank reactor (CSTR) with working volume of 6.4 L. The CSTR was made of acrylic with a gas-liquid-solid separating device. The system temperature was controlled at 37 °C by a heater. The chemical oxygen demand (COD) was maintained at 6000 mg/L^28^ by diluting the produced food waste hydrolysate during the whole continuous tests. The CSTR was operated in batch mode until biogas was produced. Thereafter, bioreactor was switched to continuous mode with HRT of 12 h until steady state condition was obtained. The CSTR was sampled at a fixed HRT over at least three days. The HRT was then decreased to the next level and the bioreactor was operated until steady state condition was achieved. Fermentation pH in the batch and continuous systems was automatically controlled above 4 by addition of 2 M NaHCO_3_ solution to eliminate the negative effects of low pH on the hydrogen-producing microorganisms.

### Analytical methods

Prior to analysis, the aqueous samples were centrifuged at 8,000 rpm for 5 min and filtered by a 0.45 μm filter. The glucose concentration produced in the food waste hydrolysate was quantified using the high performance liquid chromatography (HPLC) which was equipped with a BIO-RAD column (HPX-87H), a refractive index detector and a photodiode array analyzer. The detailed procedure of glucose analysis was described by our earlier publications^17^. COD was measured by the dichromate method using a COD analyzer (DR2800, HACH). VSS was measured according to the Standard Methods^29^.

The volume and composition of the produced biogas were daily analyzed. The volume of the produced biogas was quantified using a wet gas meter. Composition of the produced biogas was determined by using a gas chromatograph (Agilent 7890, USA) with a thermal conductivity detector (TCD) and a stainless steel column (2 m × 5 mm) filled with Porapak Q (50–80 meshes). The carrier gas was nitrogen with a flow rate of 30 mL/min. The volume of the injected sample was 0.5 mL.

The soluble microbial products (SMPs) in the fermentation samples were determined by using a gas chromatograph (Shimadzu 2010, Japan) equipped with a flame ionization detector (FID). A 2-m stainless steel column was packed with the supporter GDX-103 (60–80 meshes). The temperatures of the injection port and detector were 220 °C and 240 °C, respectively. Nitrogen was used as the carrier gas at 40 mL/min. Total SMP was calculated as the weighted sum of individual SMPs concentrations.

In this study, the hydrolysis and fermentation experiments were conducted in replicates. Average values and error bars of the duplicate experiments were shown.

## Additional Information

**How to cite this article**: Han, W. *et al*. Biohydrogen production from enzymatic hydrolysis of food waste in batch and continuous systems. *Sci. Rep.* **6**, 38395; doi: 10.1038/srep38395 (2016).

**Publisher's note:** Springer Nature remains neutral with regard to jurisdictional claims in published maps and institutional affiliations.

## Figures and Tables

**Table 1:** Comparison of glucose production from enzymatic hydrolysis of food waste by commercial glucoamylase or glucoamylase produced from solid state fermentation by fungi (*A. awamori* and *A. oryzae*).

Food waste (g)	Glucose production (g)	Glucose yield (g/g substrate)	Hydrolysis time (h)	References
80	24.35	0.304	7	This study
100	36.9	0.369	24	17

**Table 2:** Performance of soluble microbial products and carbon recoveries under steady state from food waste hydrolysate in the batch system.

Parameters	Value
Ethanol (mmol)	108.93
Acetate (mmol)	79.4
Butyrate (mmol)	31.78
Carbon dioxide (mmol)	161.67
Carbon recovered in soluble microbial products and carbon dioxide (mmol)	665.45
Consumed glucose (mmol)	132.27
Consumed carbon (mmol)	793.62
Carbon recovery (%)	83.8

**Table 3:** Comparison of hydrogen production rate obtained from this study with other reported studies.

Reactors	Substrate	HRT (h)	HPR (mmol/(h·L))	References
UASB	Glucose and xylose	12	5.4	23
Fix-bed reactor	Glucose	4	16.1	22
CSTR	Glucose	2.8	5.31	30
CSTR	Food waste hydrolysate	4	8.02	This study

**Table 4:** Soluble microbial products at different HRTs in the CSTR.

HRT (h)	Ethanol (mmol/L)	Acetate (mmol/L)	Butyrate (mmol/L)	Propionate (mmol/L)	SMP (mmol/L)
12	13.2	8.4	2.4	1.43	25.43
10	17.3	10.13	2.73	1.51	31.67
8	18.3	12.4	3.2	1.32	35.22
6	20.3	13.2	2.5	0.87	36.87
4	10.4	7.83	1.75	0.84	20.82

**Table 5:** Carbon recoveries with various hydraulic retention times from food waste hydrolysate in the CSTR.

Parameters	HRT (h)				
	12	10	8	6	4
Glucose utilization (%)	98.2	97.1	95.3	92.5	60.7
Glucose consumed (mmol/(L·d))	61.76	73.28	89.9	116.34	114.52
Carbon consumed (mmol/(L·d))	370.56	439.68	539.4	698.04	687.12
Carbon in SMPs (mmol/(L·d))	114.18	168.74	234.48	318.44	411.96
Carbon in CO_2_ (mmol/(L·d))	135.36	154.08	190.44	288.72	158.4
Carbon recovered (mmol/(L·d))	249.54	322.82	424.92	607.16	570.36
Carbon recovery (%)	67.3	73.4	78.7	86.9	83

**Table 6:** Carbon balance of biohydrogen production from enzymatic hydrolysis of food waste in the batch and continuous systems (HRT = 6 h).

Percentage (%)	Batch	CSTR
Ethanol	18.52	15.7
Acetate	13.5	10.2
Butyrate	10.81	3.87
Propionate	ND	1
Carbon dioxide	13.74	27.88
Sludge and others	10.93	8.84
Undigested solid	32.5	32.5
Total	100	100

ND = not detected by gas chromatograph.

**Table 7:** Comparison of hydrogen yield from food waste in the batch and continuous systems.

Pretreatment	microorganism	Reaction mode	Hydrogen yield (mL/g VSS_added)_	References
Sonication	No inoculum	Batch	97	31
Alkalization + ultrasonication	Sewage sludge	Batch	13.8	32
Autoclaving	*Clostridium butyricum* and *Clostridium pasteurianum*	Batch	38.9	33
Grind	Sewage sludge	Continuous	205	27
pH and temperature	Anaerobic sludge	Continuous	310	34
Enzymatic hydrolysis	Sludge	Batch	967.2	This study
Enzymatic hydrolysis	Sludge	Continuous	810.2	This study

**Table 8:** Compositions of food waste used in this study (per 100 g food waste).

Component	Value (g)	Component	Value (g)
Moisture	72.3 ± 1.5	Starch (dry basis)	40.6 ± 0.6
Total solid (TS)	28.6 ± 2.3	Protein (dry basis)	10.5 ± 0.5
Volatile solid (VS)	25.4 ± 0.9	Total phosphorus (dry basis)	1.6 ± 0.06
Carbohydrate (dry basis)	42.7 ± 0.8	Lipid	6.2 ± 0.7
